# Reclaiming of Amine CO_2_ Solvent Using Extraction of Heat Stable Salts in Liquid-Liquid Membrane Contactor

**DOI:** 10.3390/membranes13020230

**Published:** 2023-02-14

**Authors:** Sergey Shirokikh, Denis Kalmykov, Dmitry Matveev, Stepan Bazhenov

**Affiliations:** A.V. Topchiev Institute of Petrochemical Synthesis RAS, 29 Leninsky Prospekt, 119991 Moscow, Russia

**Keywords:** extraction, heat stable salts, liquid-liquid membrane contactor, alkanolamines, carbon dioxide emission, absorption

## Abstract

Amine CO_2_ solvents undergo oxidative degradation with the formation of heat stable salts (HSS). These HSS reduce the sorption capacity of amines and lead to intense corrosion of the equipment. In our work, we propose a membrane-supported liquid-liquid extraction of the HSS from alkanolamines. For this purpose, a hollow fiber membrane contactor was used for the first time. A lab-scale extraction system on the basis of a hollow-fiber liquid-liquid membrane contactor with hollow fiber ultrafiltration polyvinylidenefluoride and polysulfone membranes has been studied. The extraction of the HSS-ions from a 30 wt.% solution of monoethanolamine was carried out using a 0.25–1 M solution of OH-modified methyltrioctylammonium chloride in 1-octanol as an extractant. It has been shown that >90% of HSS ions can be extracted from the alkanolamine solvent within 8 h after extraction. The results obtained confirm the possibility of using membrane extraction with a liquid-liquid membrane contactor for the reclaiming of amine CO_2_ solvents to increase the general efficiency of carbon dioxide capture.

## 1. Introduction

One of the main environmental challenges in the climate change limitation is to limit emissions of carbon dioxide (CO_2_) into the atmosphere. Governments of various countries are taking certain actions aimed at reducing the amount of such emissions. An important part of solving this problem is the CO_2_ capture from emissions produced by industry and power plants [1,2,3]. Carbon dioxide is also an acidic component of natural gas. During the natural gas processing, CO_2_ must be removed to reduce equipment corrosion and enhance low heating value of gas [4].

CO_2_ absorption using liquid alkanolamine-based solvents is the most mature CO_2_ capture method available [5,6]. This technology has already reached the pilot and demo levels within the post-combustion CO_2_ capture area [7,8]. Aqueous solutions of monoethanolamine (MEA) and N-methyldiethanolamine (MDEA) are mainly used as liquid CO_2_ solvents [9,10]. Several novel sterically hindered and cyclic amines are also used for CO_2_ separation from flue gases. The use of such pure amines, or mixed with conventional solvents, shows some promise for improving the effectiveness of absorption processes [11,12]. The main disadvantage of the absorption technology is the degradation of solvents due to oxidation and thermal destruction of alkanolamines. This problem arises due to the presence of a significant amount of oxygen (usually 4–15 vol.%) in separated gas mixtures such as flue gases [13], and elevated temperatures (up to 140 °C) at the stage of CO_2_ desorption. Carboxylic acids, amides, amines, aldehydes, ammonia, etc. are formed as degradation products [14]. Moreover, solvent amines can also interact with gas mixture impurities (SO_x_, NO_x_, etc.) and equipment corrosion products. The interaction of alkanolammonium ions and organic or inorganic acids in solvent leads to the formation of heat stable salts (HSS). HSS do not decompose during the CO_2_ desorption step. The constant increase in the HSS content in the system during CO_2_ sorption/desorption cycles reduces the CO_2_ solvent capacity and accelerates the corrosion of the equipment [15,16].

There are several potentially applicable industrial methods for HSS removal for the reclamation of amine CO_2_ solvents. These methods include thermal distillation [17], ion exchange [18,19], and electrodialysis [20,21,22]. Comparatively new methods for extracting HSS have also been proposed: nanofiltration for preliminary concentration of HSS [23], as well as novel direct liquid-liquid extraction with hydrophobic organic extractants (Aliquat^®^ 336—ionic liquid, quaternary ammonium base dissolved in aliphatic alcohols) [24,25].

An alternative to the liquid extraction method may be to use membrane-supported extraction in liquid-liquid membrane contactors. Membrane extraction methods make it possible to extract HSS at a low concentration with minimal energy consumption. The use of liquid-liquid membrane contactors also has the following advantages: (1) no phase dispersion, as the membrane acts as a mass transfer surface; (2) modularity and compactness; (3) low metal capacity of the equipment; and (4) energy efficiency [26,27]. The possibility of using membrane contactors for extraction of ions has been shown in a number of studies [28,29,30,31].

The hollow fiber membrane contactor provides the densest packing of the membrane in the module (i.e., the largest mass transfer surface area per volume unit of the module). Hollow fibers from various widespread membrane materials such as polypropylene (PP), polysulfone (PSF), polyethersulfone (PES), polytetrafluoroethylene (PTFE), and polyvinylidenefluoride (PVDF) have been used for liquid-liquid membrane-supported extraction of carboxylic acids from aqueous phase. For example, in the work [29], PP hollow fibers were used for the extraction of biobasic 3-hydroxypropionic acid using tri-n-octylamine in n-decanol. The authors also noted the usefulness of continuous acid back-extraction to maintain an effective driving force for its transfer to the organic phase. The implemented process ensures high selectivity of extraction of the target acid, a large mass transfer surface area provided by the microporous membrane, and the absence of emulsion formation [29].

To remove carboxylic acids from such water streams as fermentation broths, commercial PTFE hollow fibers with a pore diameter of 470 nm, an outer diameter of 3.5 mm, and an inner diameter of 3 mm have been successfully used [32]. The choice of PTFE fibers is related to their chemical stability and high resistance to fouling, which allows them to be used for a long time. In the case of extraction of acids from fermentation broths, in addition to the usual advantages, such as the absence of emulsification of the contacting phases, membrane extraction reduces the toxic effect of solvents on microorganisms compared with traditional liquid extraction. Membrane extraction was carried out in PTFE hollow fiber membrane contactors with a surface area of up to 0.15 m^2^ using tri-n-octylamine in n-decanol as the extractant. The removal of lactic acid from the fermentation broths resulted in a glucose conversion rate of up to 80 mol %. At the same time, a back extraction strategy was used to improve the efficiency of the process. Accordingly, during back-extraction, a peak concentration factor of up to 7.8 could be shown [32].

The authors of the work [30] carried out a direct comparison of three different membranes made from PSF, PES, and PVDF in the process of liquid-liquid membrane-supported extraction of organic acids (formic, acetic, and propionic) from aqueous solutions. The results obtained demonstrated a significant difference in membrane performance for the same system, even though one would normally expect the membrane to only play a role in promoting a high interfacial mass transfer area. At the same time, the type of extractant and the extracted target component also had a versatile effect on the mass transfer and separation coefficients [30].

Analysis of available literature data shows that hollow fiber membrane contactors have not previously been used to remove HSS from alkanolamine CO_2_ solvents. Consequently, the purpose of this work is to demonstrate the possibility of reclaiming amine CO_2_ solvents using extraction in a hollow fiber liquid-liquid membrane contactor. In addition, in this work, we compared two types of hollow fibers in a membrane contactor for liquid-liquid membrane-supported HSS extraction. To implement this process, we used commercial polyvinylidenefluoride hollow fibers and lab-scale polysulfone hollow fibers fabricated within this work.

## 2. Materials and Methods

### 2.1. Materials and Reagents

Commercial ultrafiltration polyvinylidenefluoride hollow fibers, PVDF-300 (Faserkraft, Moscow, Russia, inner diameter—1.2 mm, outer diameter—2 mm), and polysulfone hollow fibers that were synthesized in this work were used as membranes of the liquid-liquid contactor. Polysulfone (BASF Ultrason^®^ S 6010, Ludwigshafen, Germany) was used in the form of granules in the work, as well as N-methylpyrrolidone (NMP, 99% extra pure, Acros Organics, Geel, Belgium) as a solvent for the preparation of dope solutions. Polyethylene glycol with an average molecular weight of 400 g∙mol^−1^ (PEG-400, Acros Organics, Geel, Belgium) was utilized as a pore-forming additive. All reagents were used without additional purification. Carbon dioxide and helium (MGPZ, Moscow, Russia) were used for the gas permeance measurements of hollow fibers. Monoethanolamine (LLC TD HIMMED, Moscow, Russia) was used for the preparation of the model CO_2_ solvent. Formic acid and oxalic acid dehydrate (LLC TD HIMMED, Moscow, Russia) were used as the model HSS. To remove HSS from alkanolamine solvents, 0.25–1 M solution of quaternary ammonium salt trioctylmethylammonium chloride (Aliquat^®^ 336, BASF, Ludwigshafen, Germany) in n-octanol (Sigma-Aldrich, St. Louis, Missouri, USA) was used as an extractant. To increase the efficiency of extraction, Aliquat^®^ 336 was converted into its OH-form according to the method described [24,25] until the 65% OH- degree of conversion was achieved.

### 2.2. PSF Hollow Fiber Membranes Preparation

To create PSF hollow fiber membranes, the dope solution was chosen, which was used by us earlier [33,34]. PSF and PEG-400 (mass ratio 1:1.36) were placed in a thermostatically controlled reactor and stirred at a speed of 150 rpm at a temperature of 50 °C. NMP was then added to this system, while increasing the stirring speed to 500 rpm. Under these conditions, the solution was mixed until homogeneity was achieved. The concentration of PSF in the dope solution assumed a value of 22 wt.%. Before hollow fiber membrane spinning, the polymer solution was filtered under a nitrogen pressure of 1.8–2.0 bar through a stainless-steel mesh (cutoff rating of 4–5 μm). After the filtration procedure, the polymer solution was cooled to room temperature and degassed under vacuum overnight.

Before hollow fiber membranes preparation, the dynamic viscosity of the dope solution was determined using a Brookfield viscometer Brookfield DV2T-RV (AMETEK Brookfield, Middleboro, MA, USA). Samples of PSF hollow fiber membranes were prepared via a wet spinning mode using the setup described in another study [35]. After spinning, the samples of hollow fiber membranes were sequentially washed with tap water, then with ethanol for 2 h, and finally with n-hexane for 2 h, after which they were dried in air at room temperature.

### 2.3. Membrane Characterization

To study the structure and the main transport properties affecting the membrane-supported extraction process, PVDF-300 and PSF hollow fiber membranes were characterized by a number of standard techniques. The pore size was measured by liquid–liquid displacement porosimetry using the porometer POROLIQ 1000 ML (Porometer, Ghent, Belgium). Membrane pore size analysis was performed via a liquid-liquid displacement method using water-saturated isobutanol and isobutanol-saturated water as a solvent pair. The displacement of the wetting liquid was carried out by a stepwise increase of the transmembrane pressure. The flow through the membrane was measured after 180 s of the initial stabilization time at each applied pressure. The measurements were stopped after the complete displacement of the wetting liquid was achieved. The technique allows the measurement of the pore size distribution from 2 to 500 nm. The size was calculated according to the procedure described in detail [36]. The porous structure was characterized by the mean flow pore size. The mean flow pore size value is defined as the pore size at which 50% of the flux penetrates through the larger pores and 50% of the flux penetrates through the smaller pores of the membrane skin layer.

The gas transport properties of PVDF-300 and PSF hollow fiber membranes were studied by the volumetric method using the individual gases helium and carbon dioxide. Differences in molecular mass between the chosen gases make it possible to reliably establish the presence of the Knudsen flow regime by the ratios of the permeances for individual gases (i.e., ideal selectivity) [37]. This procedure will indirectly confirm the results of porosimetry and additionally characterize the porous structure of the membranes. The gas permeance (*P*/l) was calculated according to the Equation (1):

*P*/l = Q/(Δp·S), (1)

where (*P*/l) is the gas permeance of individual gas, m^3^/(m^2^·h·atm) [38]; Q is the volumetric flow rate of the gas passed through the membrane, m^3^/h; Δp is the transmembrane pressure, atm; and S is the membrane active surface area, m^2^. The gas volume passing through the membrane from the inner cavity was measured using a Shinagawa DC-1 dry gas meter (Shinagawa, Tokyo, Japan) at room temperature (23 ± 2 °C) at a transmembrane pressure between 0.5 and 2 bar, while the permeate pressure was kept constant at 1 bar. The ideal selectivity, α, for the He/CO_2_ gas pair was calculated using Equation (2):

α = (*P*/l)_He_/(*P*/l)_CO_2__, (2)

where (*P*/l)_He_ is the individual permeance of the helium, m^2^/(m^2^·h·atm), and (*P*/l)_CO_2__ is the individual permeance of the carbon dioxide, m^2^/(m^2^·h·atm).

The membrane structure and morphology were investigated via scanning electron microscopy (SEM), using a Thermo Fisher Phenom XL G2 Desktop SEM (Thermo Fisher Scientific, Waltham, MA, USA). The samples were broken in liquid nitrogen after preliminary impregnation of the fibers in isopropanol, and a layer of gold was applied to them using a Cressington 108 auto Sputter Coater (Cressington Scientific Instruments, Watford, Great Britain) desktop magnetron sputtering machine. The thickness of the gold layer was 5–10 nm. Micrographs were obtained at an accelerating voltage of 15 kV.

### 2.4. Lab-Scale Extraction Experiments

The membrane-supported extraction of HSS from alkanolamine solvents was performed using a self-made liquid-liquid membrane contactor system. To build a contactor system PVDF or PSF hollow fibers were placed inside a glass tube and its ends were sealed with epoxy resin. Table 1 contains the geometrical properties of the membrane contactor.

**Figure 1:**
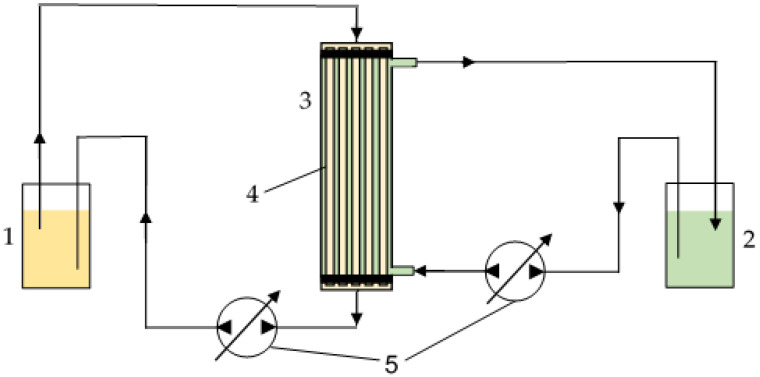
The scheme of a lab-scale membrane-supported extraction process: 1—a vessel with OH-modified Aliquat^®^ 336 solution in n-octanol; 2—a vessel containing MEA solution; 3—a membrane contactor; 4— PVDF-300 membranes; and 5—peristaltic pumps.

Model alkanolamine solvents and extractant were prepared gravimetrically. A 30 wt.% MEA solution containing about 2360 mg/L HSS [21] and a solution of OH-Aliquat^®^ 336 in n-octanol were supplied to the contactor in counter-current mode using peristaltic pumps. The extractant was fed from the lumen side of the fiber and the MEA solvent was fed from the shell side of the membrane. The liquid flow was carried out in laminar mode (*Re* = 140) with a linear flow rate of phases varied within 0.5–0.8 cm/s. To smooth out potential local concentration fluctuations that could affect the sampling process, magnetic anchors were placed in the vessels with used liquids and placed on the magnetic stirrers (C-MAG HS 7, IKA, Staufen, Germany). The 30 wt.% MEA solution containing about 2360 mg/L HSS and a solution of OH-Aliquat^®^ 336 in n-octanol in the vessels were stirred at a speed of 200 rpm. This mode was set according to preliminary experiments to establish the optimal process parameters. With an increase or decrease in the difference in the linear flow rate of the phases, dispersion of the MEA solution and the extractant solution was observed. The scheme for the process of membrane-supported HSS extraction from alkanolamine solvents using a liquid-liquid membrane contactor is shown in Figure 1.

The efficiency of the membrane-supported extraction process was indicated by HSS concentration change in the MEA solution, which was monitored every hour using an ion chromatography method. In this case, a sample of 1–2 mL of the MEA solution was used. The concentration of HSS in MEA solutions was determined as formic or oxalic acid anion concentration using the ionic chromatograph (“Akvilon Stayer-Ì”, chromatographic column Shodex ICSI-50 4E, eluent—3.2 mmole NaHCO_3_ and 0.1 mmole Na_2_CO_3_) equipped with the electromembrane suppressor EMCES 21, and conductometric detector CD-510 (JSC “Akvilon”, Podolsk, Russia). The error in determining the HSS ion concentration was not greater than 3%. For a more detailed comparison of the efficiency of the membrane-supported extraction process under different conditions, the concentration of the HSS ions in the MEA solution was calculated as C_t_/C_0_, where C_t_ is the concentration of HSS ions at a given time, and C_0_ is the initial concentration of HSS ions.

## 3. Results and Discussion

### 3.1. PVDF and PSF Hollow Fibers Characteristics

In addition to the direct interest in studying the structure of the obtained PSF hollow fibers and their comparison with commercial PVDF fibers, characterization of the membranes may be of further interest. In our previous work [39], it was shown that the porous structure of the membrane can be changed during contact with an alkanolamine CO_2_ solvent and the OH-Aliquat^®^ 336 solution in n-octanol. Changes in the membrane morphology and porous structure can affect the membrane-supported liquid-liquid extraction process and provide explanations for some of the observed effects. In addition, the use of commercial membranes requires confirmation of their properties and study of the porous structure for further correct comparison using alternative technologies, and monitoring of changes during the extraction process.

**Figure 2:**
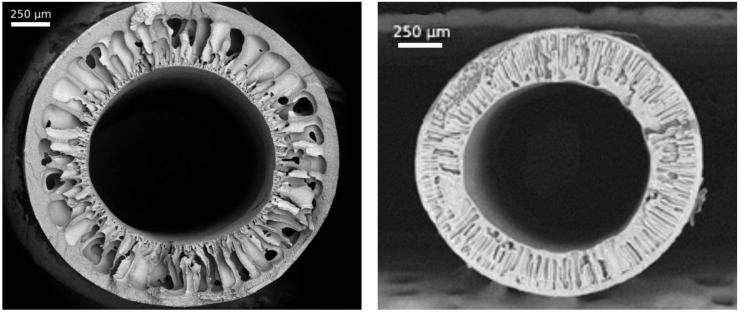
SEM images of cross-sections of a PVDF hollow fiber (**a**) and a PSF hollow fiber (**b**).

Figure 2 presents cross-sectional SEM images of a used commercial PVDF membrane and a fabricated PSF hollow fiber membrane. Evidently, both types of membranes have an asymmetric structure with a thin selective layer and the porous support pierced with finger-like macrovoids.

By analysis of the SEM images, the geometrical parameters of the PSF hollow fiber, such as average outer and inner diameters, were estimated. The results obtained are shown in Table 1.

The data of PVDF and PSF hollow fiber porosimetry and permeance measurements are summarized in Table 2.

PVDF hollow fiber membranes have a mesoporous structure of the skin layer. This is evidenced by the results of the pore size, the values of gas permeance, and ideal selectivity (α = 2.23 for He/CO_2_ gas pair). Also, a thin layer of PSF membranes has a mesoporous structure with a smaller pore size. The permeance of the PSF hollow fiber membrane for individual gas CO_2_ is 2.22 m^3^/(m^2^·h·atm), while selectivity α(He/CO_2_) is 2.94. It should be noted that the values of the ideal selectivity for the He/CO_2_ gas pair indicate that in the obtained membranes, gas transport appears to be close to the Knudsen gas flow regime which occurs in pores with sizes of 2 to 50 nm. Thus, in the Knudsen gas flow regime, the ideal selectivity for the He/CO_2_ gas pair is 3.3. At the same time, the results of porosimetry show that the average pore size of the skin layer on the outer surface of the PSF membrane is 10 nm and the average pore size of the PVDF membrane is 43 nm. However, the obtained data may also indicate the existence of a viscous gas-flow regime. This may confirm the existence of a wide distribution of pore sizes in a thin porous selective layer of membranes and the existence of pores with a size significantly exceeding the boundary of the mesoporous sizes (50 nm). However, it should be noted that despite the presence of large pores in the structure of the selective membrane layer, no phase dispersion was observed during the entire extraction study.

### 3.2. Extraction of HSS in Liquid-Liquid Hollow Fiber Membrane Contactor

During the extraction process, the HSS ions diffuse through the pores of the membrane and interact with the extractant according to the overall equation:

$$R_{4}N^{+}{\mathrm{OH}}^{-}+{\mathrm{HCOO}}^{-}⇋R_{4}N^{+}{\mathrm{HCOO}}^{-}+{\mathrm{OH}}^{-},$$

where R = C_8_H_17_ and CH_3_, when using OH-modified Aliquat^®^ 336.

**Figure 3:**
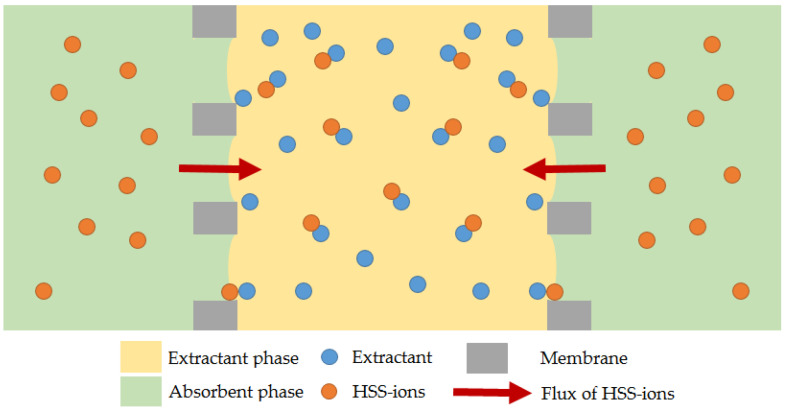
The diagram of the HSS ions transport process during membrane-supported extraction in the liquid-liquid membrane contactor.

For better clarity, Figure 3 shows the diagram of the HSS ion transport process during membrane-supported extraction in the liquid-liquid membrane contactor.

Since the PVDF membrane is completely wetted with MEA solution [39], the porous structure of the membrane is likely to be filled with the MEA solution with HSS ions, and the mass transfer surface will be close to the extractant solution or within the pores close to the organic phase. At the same time, when using PSF membranes, their porous structure will be more filled with OH-modified Aliquat^®^ 336 solution in n-octanol. The mass transfer surface in this case will be closer to the water phase.

**Figure 4:**
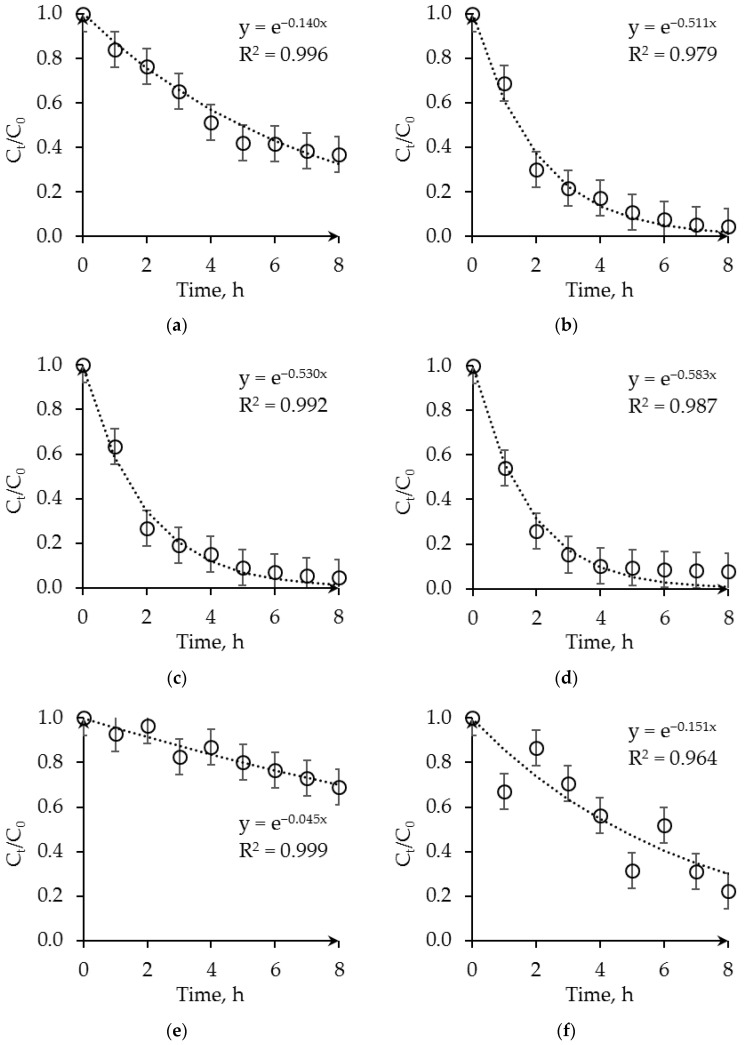
The dependence of the relative concentration of HSS ions in the MEA solution on the extraction time: PVDF fibers—(**a**–**d**), PSF fibers—(**e**,**f**); (**a**,**e**) model HSS is formate; 0.25 M OH-modified Aliquat^®^ 336 in n-octanol; (**b**) model HSS is formate; 0.5 M OH-modified Aliquat^®^ 336 in n-octanol; (**c**,**f**) model HSS is formate; 1 M OH-modified Aliquat^®^ 336 in n-octanol; (**d**) model HSS is oxalate; and 1 M OH-modified Aliquat^®^ 336 in n-octanol. C_t_—concentration of HSS ions at a given time, C_0_—initial concentration of HSS ions.

Using the laboratory system described in Section 2.3, we studied the effect of various parameters on the membrane-supported extraction of HSS from alkanolamine CO_2_ solvents. Figure 4 shows the time dependencies of the HSS ions content in the 30 wt.% MEA solution when using different concentrations of OH-modified Aliquat^®^ 336 solution in n-octanol, the type of HSS anion, and type of fibers (PVDF or PSF).

**Figure 5:**
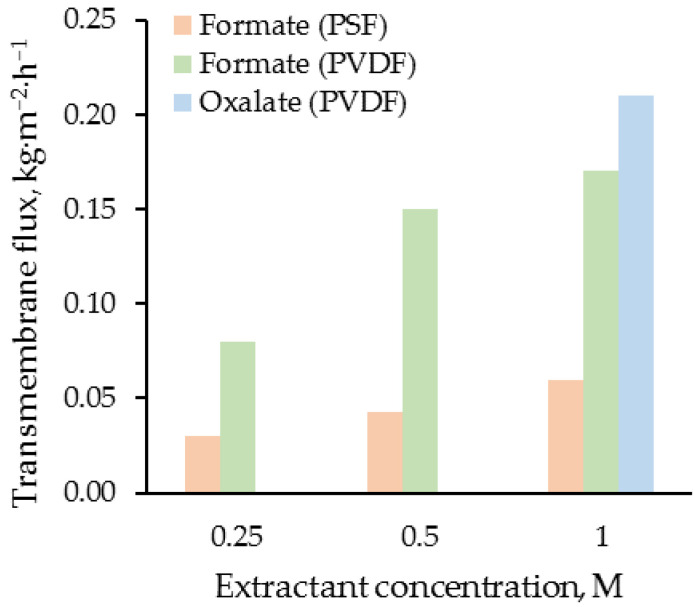
The dependence of the HSS ions flux through the PVDF-300 and PSF membranes on the OH-modified Aliquat^®^ 336 concentration and ions type.

It can be seen that there are general trends when using both PVDF and PSF fibers in terms of changing the efficiency of the extraction process depending on the concentration of the extractant. Depending on the concentration of OH-modified Aliquat^®^ 336, the membrane-supported extraction of HSS ions from alkanolamine CO_2_ solvents proceeds at different rates. The dependences of the concentration of HSS ions on time in the MEA solution practically do not differ when the extractant concentration is 0.5–1.0 M. At a OH-Aliquat^®^ 336 concentration of 0.25 M, the rate and degree of extraction of HSS ions are much lower. At the same time, the velocity of extraction of HSS ions from the 30 wt.% MEA solution when using PSF fibers is much lower than when using PVDF fibers. The results obtained can be explained by a smaller mass transfer surface in the case of using PSF fibers with their lower surface porosity, as well as a higher resistance to mass transfer due to the lower pore sizes. This is confirmed by the data in Figure 5, which shows the flux of the HSS ions through the membrane. At the same time, the replacement of monocarboxylic formic acid with dicarboxylic oxalic acid, which is a more active corrosive product of the destruction of amines, does not lead to a noticeable change in the rate and degree of extraction of HSS ions. These results demonstrate the potential to extract various HSS ions without concentrating the specific ion.

Since the extractant is taken in excess (HSS ions molar concentration is 0.025–0.050 M), the ion exchange reaction between the extractant and HSS ions can be considered as a first order reaction and described using an exponential function. The found values of effective interaction rate constants increase with OH-modified Aliquat^®^ 336 concentration growth. In the case of PVDF fibers and of 1 M OH-modified Aliquat^®^ 336 solution, the effective interaction rate constants were 0.5–0.6 h^−1^ for both formate and oxalate ions. At the same time, the rate of extraction of oxalate ions was slightly higher. A smooth decrease in the HSS concentration also indirectly indicates the absence of changes in the porous structure of the PVDF-300 membranes during their 8-h contact with the MEA and extractant solutions. The observed effect additionally confirms the applicability of the selected PVDF membranes for the process under study. Therefore, with a large excess of extractant, more than 90% of the HSS ions were extracted from 30 wt.% MEA solutions over 8 h using the membrane-supported extraction system with PVDF fibers. At the same time, when using PSF fibers, the effective interaction rate constants were significantly lower (~0.15 h^−1^) even at high concentrations of the extractant. For 8 h of the process with this configuration of the module, it was possible to extract up to 78% of HSS anions from the used amine solvent. The results obtained in the case of PSF fibers did not give a sufficient indicator in comparison with alternative processes. The flux of HSS ions for the first hour of the experiment through the PVDF-300 and PSF membranes was also calculated. Figure 5 presents the flux of the HSS ions through the membrane vs. OH-modified Aliquat^®^ 336 concentrations in extractant by ions type.

With an increase in the OH-modified Aliquat^®^ 336 concentration, the flux of HSS ions from the MEA solution into the extractant increased non-linearly, both in the cases of PVDF hollow fibers and PSF hollow fibers. The data obtained may indicate that there is a limiting effective concentration of the extractant (≥0.5 M), which allows the ion exchange reaction between the OH-Aliquat^®^ 336 and HSS ions to proceed on the mass transfer surface close to the stoichiometric ratio. The difference between the obtained characteristics of the lab-scale liquid-liquid membrane-supported extraction when using PSF and PVDF hollow fibers correlates with the results of the structure and transport properties investigation of membranes (Table 2). The lower transmembrane flux of HSS ions through the PSF membrane can be explained by the smaller pore size and, apparently, lower surface porosity. At the same time, the ion-exchange reaction between dicarboxylic oxalic acid and the extractant proceeds more intensively in comparison to that of formic acid. This effect is consistent with the literature data, and confirms the previously described mechanisms of interaction between the HSS ions and OH-Aliquat^®^ 336 [24,25].

To demonstrate the potential of the results obtained, membrane supported liquid-liquid extraction was also compared with alternative methods for removing the HSS ions from alkanolamine solvents (Table 3).

As can be seen from the table presented, most of the alternative methods can potentially be implemented in practice only in the case of high energy costs to create a high transmembrane pressure or voltage. Some of the processes presented are implemented in batch mode, requiring additional regeneration steps or subsequent separation of the mixed phases in settling tanks. At the membrane-supported extraction of the same time, the HSS ions in liquid-liquid membrane contactors ensure effective contact of the alkanolamine solvent and extractant during a continuous process. It provides a high potential for the implementation of this technology.

## 4. Conclusions

In this work, a lab-scale membrane-supported extraction system was developed for the reclaiming of amine CO_2_ solvents. It can be seen that the removal of HSS ions from aqueous amine solvents is fundamentally possible for all the membranes studied, with the most efficient membrane being made of PVDF. In this case, both direct phase dispersion and the formation of a stable emulsion, which is observed during direct contact of the MEA solution with the extractant, are absent. In the case of using PSF hollow fibers, up to 78% of HSS ions can be removed in 8 h of the liquid-liquid membrane-supported extraction process, which is not a completely satisfactory result. In turn, it is possible to extract more than 90% of the HSS ions from 30 wt.% MEA solution in 8 h using PVDF hollow fiber membrane contactor system using OH-modified Aliquat^®^ 336 in n-octanol as extractant. The results obtained demonstrate the possibility of the membrane-supported extraction process to reclaim amine CO_2_ solvents and increase the overall efficiency of the carbon dioxide capture process. However, to successfully implement this process in practice, it is necessary to develop an extractant regeneration system and scale up the technology to the level of pilot plants. It is also necessary to provide a detailed study of the extraction process using real mixtures of HSS in alkanolamines and the influence of the technological parameters of the process on the efficiency of its extraction.

## Figures and Tables

**Table 1:** Geometrical properties of the membrane contactor with PVDF-300 and PSF hollow fibers.

Parameter		Value
Active fiber length, m		0.15
Inner diameter of fiber, mm	PVDF	1.2
	PSF	1.2
Outer diameter of fiber, mm	PVDF	2.1
	PSF	1.6
Shell inner diameter, m		0.015
Membrane surface area, cm^2^	PVDF	50
	PSF	40
Packing density, m^2^/m^3^		200

**Table 2:** Characteristics of PVDF-300 and PSF hollow fiber membranes.

**Material**	PVDF		PSF	
**Type**	asymmetric		asymmetric	
Pore size *, nm	43 ± 5		10 ± 2	
***P*/l, m^3^(STP) (m^2^∙h∙atm)^−1^**	He	1430 ± 20	He	6.53 ± 0.10
	CO_2_	640 ± 10	CO_2_	2.22 ± 0.10
**α (He/CO_2_)**	2.23		2.94	

* liquid–liquid displacement porosimetry.

**Table 3:** Comparison of the efficiency of the HSS ions removal from alkanolamine solvents.

Process	HSS Removal, %	Main Disadvantages	Ref.
Membrane-supported liquid-liquid extraction	90–95	Technology not yet scaled up. Difficulty in controlling the transmembrane pressure to ensure the absence of mixing of the phases	this work
Liquid-liquid extraction	88–90	Phase dispersion with long phase separation time	[24]
Nanofiltration	75–80	The need for high pressure	[20]
Thermal distillation	80–95	High energy consumption and amine losses	[17]
Ion exchange	95–97	Multistage periodic process with large volumes of regeneration chemicals	[18]
Electrodialysis	90–95	High energy consumption and amine losses at low HSS concentration	[21]

## Data Availability

The data presented in this study are available on request from the corresponding author.
